# Tailored Core‐Shell Nanocarrier for Therapeutic Drug Delivery via Visible Light Activation

**DOI:** 10.1002/anie.202514317

**Published:** 2025-10-30

**Authors:** Deric Andrade‐Alarcón, Víctor de la Asunción‐Nadal, Gastón A. Crespo, María Cuartero

**Affiliations:** ^1^ UCAM‐SENS Universidad Católica San Antonio de Murcia UCAM HiTech Avda. Andres Hernandez Ros 1 Murcia 30107 Spain; ^2^ Department of Chemistry KTH The Royal Institute of Technology Teknikringen 30 Stockholm SE‐100 44 Sweden

**Keywords:** Conductive polymers, Drug encapsulation, Heterojunction, Ion‐selective membranes, Nanoparticles

## Abstract

We present a novel drug delivery nanocarrier consisting of 300 nm‐sized POT/Fe_3_O_4_/TRZ^+^TPB^−^ nanoparticles (NPs). The mechanism is based on the (photo)oxidation of poly(3‐octylthiophene‐2,5‐diyl) (POT) (from POT^0^ to POT^+^), which facilitates the release of the positively charged drug trazodone (TRZ^+^) encapsulated in the NPs. The primary factor guiding the overall process is the maintenance of the electroneutrality condition in each NP. The incorporation of a Fe_3_O_4_ element enables the formation of an organic‐inorganic heterojunction (Fe_3_O_4_/POT) in the core of the NP. This heterojunction permits us to utilize visible light to induce the POT photooxidation to trigger the release of TRZ^+^, unlike other platforms based on a more energetic illumination requirement. The developed nanocarrier allows for a controlled drug release, achieving doses of 3.5 µg mL^−1^ of TRZ under 530 nm irradiation, 2.4 µg mL^−1^ at 625 nm, and 1.6 µg mL^−1^ at 470 nm within 1 h. The delivery is tested in undiluted blood serum, achieving an efficiency exceeding 90%. Overall, the integration of magnetic properties with a conductive polymer along with the adjustment of the band gap to enhance photooxidation performance in the visible range, constitute the conceptual innovation behind the controlled drug delivery system here presented, with TRZ as a model.

## Graphical abstract

A core‐shell nanocarrier activated by visible light, composed of poly(3‐octylthiophene‐2,5‐diyl) (POT), iron oxide (II, III) (Fe_3_O_4_), polyvinyl alcohol (PVA), and trazodone tetraphenylborate (TRZ^+^ TPB^−^). During POT photooxidation (POT^+^), TRZ^+^ is liberated driven by the maintenance of particle's electroneutrality. The inorganic‐organic POT/Fe_3_O_4_ heterojunction facilitates accurate dosage under mild irradiation, demonstrating a novel conductive polymer‐based delivery platform. F_POT_ and F_TRZ_: fluorescence of POT and TRZ.

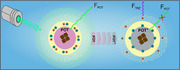

## Introduction

Systems designed for the controlled release of pharmaceuticals have attracted considerable interest owing to their capacity to modulate dosages, thereby facilitating personalized treatments with specific spatiotemporal attributes.^[^ ^1^, ^2^ ^]^ Moreover, therapeutic efficacy has claimed to be enhanced while mitigating adverse side effects associated with certain therapies, because of the capability of reducing fluctuations in medication concentration.^[^ ^3^ ^]^ A search in the literature reveals multiple platforms demonstrated for controlled drug release, including polymer‐based systems,^[^ ^4^ ^]^ lipid structures,^[^ ^5^ ^]^ hydrogels,^[^ ^6^ ^]^ and micellar systems.^[^ ^7^ ^]^ These platforms vary significantly in size, encompassing macroscopic structures used in implants, microscale particles in suspension, and nanoscale systems.

Nanocarriers are defined as engineered nanoscale systems designed to encapsulate and release therapeutic agents in a controlled manner. These have developed into viable solutions for drug delivery due to their unique properties. Because of the nanometric dimension, nanocarriers exhibit enhanced circulation within the body and the ability to traverse biological barriers, facilitating targeted drug release at specific sites.^[^ ^8^ ^]^ Their high surface‐to‐volume ratio improves drug‐loading capacity respect to other described systems. This property, combined with advanced chemical functionalization and specialized coatings, further expands their applicability options.^[^ ^9^, ^10^, ^11^ ^]^ Encapsulating certain bioactive molecules, including doxorubicin,^[^ ^12^ ^]^ testosterone,^[^ ^13^ ^]^ and paclitaxel,^[^ ^14^ ^]^ within nanocarriers has demonstrated the benefit of preventing undesirable interactions with other species, optimizing hence treatment efficacy. This strategy ensures a higher proportion of the drug reaches the target site while reducing the formation of toxic metabolites and prolonging the drug's half‐life.

To further enhance the functionality of nanocarriers, external stimuli‐responsive approaches, such as the application of magnetic fields,^[^ ^15^ ^]^ light,^[^ ^16^ ^]^ or chemical gradients,^[^ ^17^ ^]^ have been investigated aiming to trigger drug release with high precision. A notable strategy involves the use of semiconductors materials, leveraging their ability to generate electrons (e^−^) and holes (h^+^) on‐demand and in‐situ. The formation of these species (e^−^, h^+^) can be initiated using light. Then, the energy necessary to commence the drug release process can be customized by the materials composing the nanocarrier, aligning the bandgap of the created composites with the operational requirements.^[^ ^18^ ^]^ For example, inorganic semiconductors, such as titanium dioxide (TiO_2_),^[^ ^19^, ^20^ ^]^ zinc oxide (ZnO)^[^ ^21^ ^]^ and silicon‐based nanomaterials,^[^ ^22^ ^]^ have widely been explored in photocatalytic drug activation. Polymeric semiconductors, including polypyrrole (PPy),^[^ ^23^ ^]^ polyaniline (PANI)^[^ ^24^ ^]^ and poly(3,4‐ethylenedioxythiophene) (PEDOT)^[^ ^25^ ^]^ are of interest due to their tunable optoelectronic properties and biocompatibility. Moreover, semiconductors exhibit specific properties that make them very suitable for biomedical applications, e.g., photothermal heating^[^ ^26^ ^]^ and the generation of reactive oxygen species.^[^ ^27^ ^]^ Given these promising properties, ongoing research continues to explore novel semiconductor‐based materials with improved performance for drug release applications.

Polythiophene‐based nanoparticles (NPs) have recently gained attention due to their distinctive electronic properties and potential applications in encapsulation and release.^[^ ^28^ ^]^ From a fundamental point of view, poly(3‐octylthiophene‐2,5‐diyl) (POT) has extensively been studied in reversible ion‐transfer processes across polymeric membranes. In essence, POT can be oxidized from its neutral state to the charged POT^+^ and, with the needed doping assisted by the anion present in a lipophilic ion exchanger (e.g., sodium tetrakis [3,5‐bis(trifluoromethyl)phenyl] borate, Na^+^TFPB^−^), and the counter cation being expelled to balance the charges.^[^ ^29^, ^30^, ^31^, ^32^, ^33^ ^]^ The selectivity of such a cation transfer can be achieved through the incorporation of a specific receptor known as ionophore. Overall, a dynamic equilibrium is established, which can be displaced on demand towards the release or capture of the target ion. Ion‐selective POT‐based NPs have been developed following this operational principle: a functional POT core and a membrane‐like shell containing an ionophore and ion‐exchanger facilitate the reversible encapsulation and release of potassium ion (K^+^) through a light‐induced process.^[^ ^34^ ^]^

In this context, POT‐membrane NPs have been engineered in the present work to facilitate the release of complex cationic species of pharmacological relevance, exemplified herein by trazodone (TRZ). Beyond its clinical interest, TRZ was selected as a proof‐of‐concept molecule owing to its cationic and amphiphilic nature, well‐defined therapeutic plasma levels, rapid kinetic profile, and strong, easily quantifiable spectroscopic signals (UV–vis and fluorescence). These features make TRZ an experimentally robust model drug for evaluating new release systems, while the platform itself can be extended to other charged therapeutics in the future. To strengthen the performance and versatility of the former POT‐membrane NPs, a Fe_3_O_4_ element is integrated in the form of a sub‐core in the POT part, specifically demonstrating a tangible improvement in drug loading efficiency, stability and release kinetics. Truly, the effective incorporation of Fe_3_O_4_ in a conductive polymer lattice represents a transformative advancement in both optoelectronic materials and nanocarrier systems. On one hand, Fe_3_O_4_ NPs exhibit notable magnetic properties that facilitate energy level modulation and improve charge transport in composite systems. On the other hand, when combined with conductive polymers, e.g., poly(3‐hexylthiophene),^[^ ^35^, ^36^ ^]^ poly(3‐octylthiophene)^[^ ^37^ ^]^ and polyaniline,^[^ ^38^ ^]^ these properties are significantly enhanced, leading to improved conductivity, energy band alignment, and superior optoelectronic performance. Overall, a multifunctional material has been developed through a rational design approach, demonstrating strong capabilities for light‐induced and electrochemically controlled release of therapeutic agents, with TRZ as an example.

## Results and Discussions

### Preparation and Characterization of POT/Fe_3_O_4_/TRZ^+^TPB^−^ NPs

The POT/Fe_3_O_4_/TRZ^+^TPB^−^ NPs were synthesized using a one‐step method. A dispersion of POT, Fe_3_O_4_ NPs and TRZ^+^TPB^−^ in THF was added into an aqueous solution of 1% PVA under stirring (∼600 rpm). Fe_3_O_4_ NPs were used as a seeding agent in the emulsion formation process. Then, the synthesized POT/Fe_3_O_4_/TRZ^+^TPB^−^ NPs were washed by magnetic separation, removing the leftover TRZ^+^TPB^−^ that was added in excess and the non‐magnetic NPs. This process ensures the introduction of the magnetic NPs in the POT core during the nanodroplets formation. All the utilized materials and details on the synthesis procedures are provided in the Supporting Information (Section 1).

**Figure 1:**
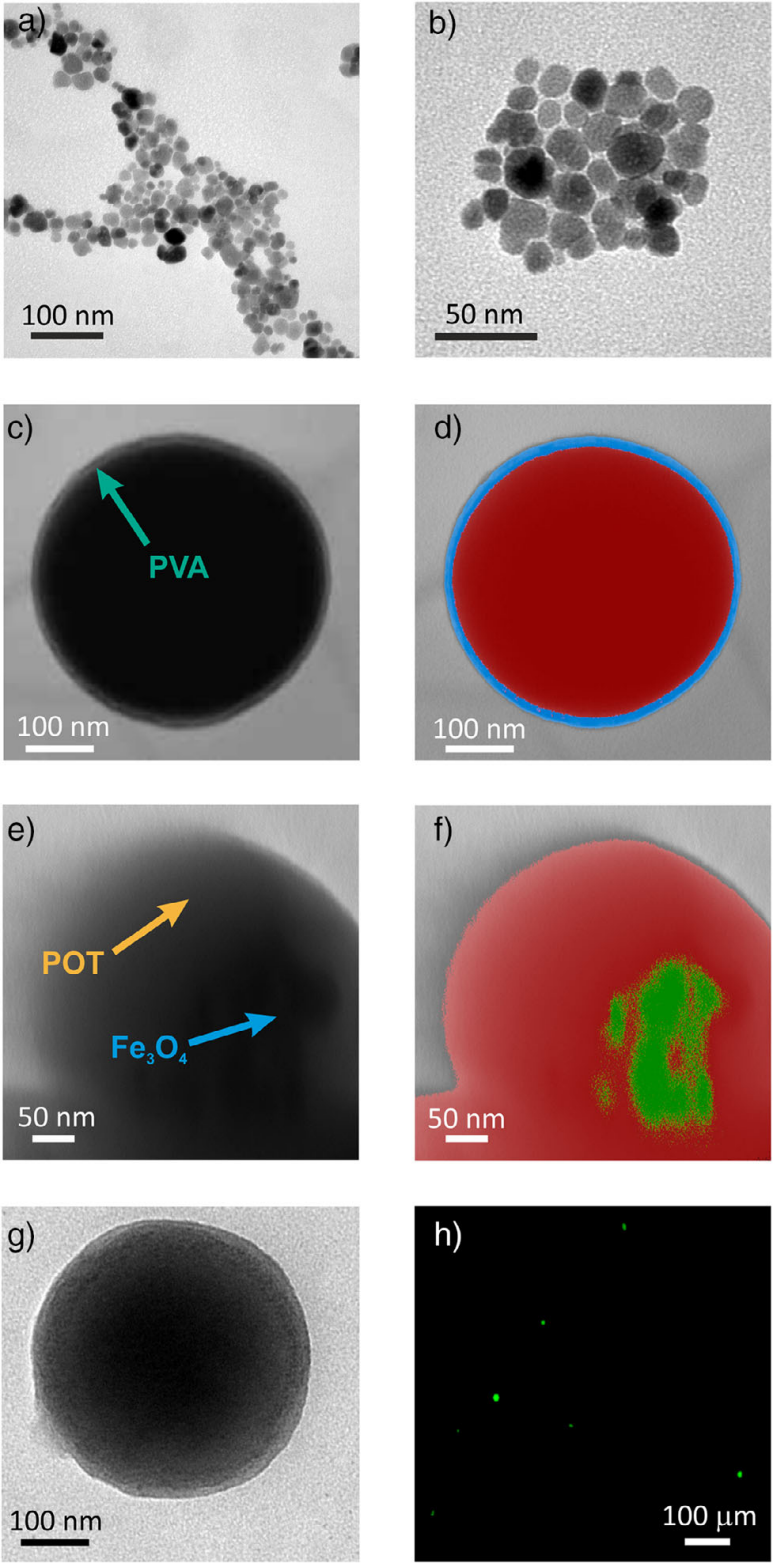
a) TEM image of Fe_3_O_4_ NPs. b) Close‐up TEM image of the Fe_3_O_4_ NPs. c) TEM image of POT/Fe_3_O_4_ NPs. d) Digitally colored remapping of the former TEM image. e) Close‐up TEM image of POT/Fe_3_O_4_ NPs. f) Digitally colored remapping of the former TEM images. g) TEM image of a POT/ Fe_3_O_4_/TRZ^+^TPB^−^ NP. h) Fluorescence Microscope Image of POT/ Fe_3_O_4_/TRZ^+^TPB^−^ NP suspension at 550 nm.

To characterize the developed NPs, transmission electron microscopy (TEM) images and the related data analysis were performed. Figure 1a,b show the characteristic structure of Fe_3_O_4_ NPs^[^ ^39^ ^]^: an inverse cubic spinel crystal structure. Further measurements confirmed the successful preparation of Fe_3_O_4_ NPs by a coprecipitation method, with a suitable average size and dispersion (14 ± 7 nm). The composition of the nanoparticles was confirmed by X‐ray diffraction (XRD) measurements, identifying the main crystallographic planes reported in the reference diffractogram (Section 2, Figure S1A). Figure 1c displays the POT/Fe_3_O_4_ NPs, revealing a spherical form with an average size of 280 ± 80 nm. The image was digitally colored according to the relative intensity of the different materials (Figure 1d) to facilitate the analysis of the shell. It is composed of PVA, with this polymer acting as a stabilizing agent of the NPs by permitting the formation of nanodroplets that will further contain the lipophilic components of the NPs. The thickness of the PVA layer is 12 ± 3 nm, which is in line with other already published reports.^[^ ^28^ ^]^ The presence of PVA in the final composite was confirmed by FTIR analysis (Section 2, Figure S1B). Close‐up images were taken to evaluate the Fe_3_O_4_ core. In Figure 1e, Fe_3_O_4_ particles are intuited to be embedded in the POT core. The different materials were colored according to their relative intensities for a better understanding (Figure 1f). Although the contrast in the TEM image may suggest a heterogeneous distribution of Fe_3_O_4_ within the polymer matrix, this is likely an artifact caused by the sensitivity of the polymer to the electron beam. The homogeneous magnetic response of the nanocomposites, the red shift of POT bandgaps upon Fe_3_O_4_ incorporation, and the absence of Fe_3_O_4_ particles decorating the NP surface confirmed indeed that the magnetic NPs are effectively embedded within the polymeric core. Effectively, the presence of NPs with similar size and geometry than the raw Fe_3_O_4_ particles was noted in the core of the POT/Fe_3_O_4_ NPs. Figure 1g depicts a POT/Fe_3_O_4_/TRZ^+^TPB NP with an identical spherical shape and dimensions as those for POT/Fe_3_O_4_ NPs, indicating that the introduction of the complex TRZ^+^TPB^−^ does not affect the shape and the size of the nanocarrier. Dynamic Light Scattering (DLS) confirmed that the NPs are well dispersed in aqueous solution, with a narrow size distribution and nearly neutral surface charge (see Section 2, Figure S1C and Table S1).

Another approach used to characterize the POT/Fe_3_O_4_/TRZ^+^TPB NPs was fluorescence spectroscopy. As shown in Figure 1h, the NPs exhibited a fluorescence emission upon excitation at 550 nm, confirming the presence of POT within the NP structure. The number of POT/Fe_3_O_4_/TRZ^+^TPB^−^ NPs in the created suspension was estimated by counting the NPs in fluorescence optical microscopy images, being the number of NPs 200 ± 20 million mL^−1^ after the preparation and washing processes. This concentration was used for the following drug‐release experiments. Moreover, the magnetic properties of the fluorescent POT/Fe_3_O_4_/TRZ^+^TPB^−^ NPs can be realized in Video S1. In the video, the NPs displayed simultaneous fluorescence and magnetic responsiveness. Upon application of an external magnet near the dispersion, it can be distinguished how the fluorescent yellow NPs move towards the magnetic source, supporting the successful integration of both fluorescent and magnetic properties.

### Mechanism Underlaying the Release of TRZ^+^ from POT/Fe_3_O_4_/TRZ^+^TPB^−^ NPs

**Figure 2:**
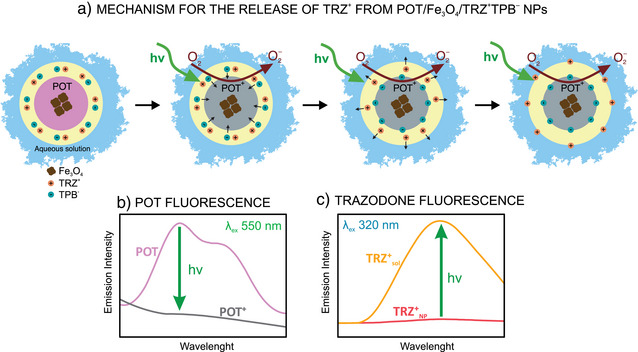
a) Mechanism underlaying the release of TRZ^+^ form POT/Fe_3_O_4_/TRZ^+^TPB^−^ NPs upon green light‐induced stimulation. POT^0^ (pink core), POT^+^ (gray core) b) Illustration of the fluorescence decrease of POT spectrum upon green light‐induced actuation. c) Illustration of the increasing fluorescence spectrum in the supernatant of TRZ^+^ after green light‐induced actuation. POT = poly(3‐octylthiophene‐2,5‐diyl), TRZ^+^=trazodone, TPB^−^=tetraphenylborate, PVA = polyvinyl alcohol, Fe_3_O_4 _= iron oxide (II, III) magnetic nanoparticles.

Figure 2a illustrates the mechanism underlaying the TRZ^+^ release upon illumination of the POT/Fe_3_O_4_/TRZ^+^TPB^−^ NPs. Notably, each component in the NP was carefully rationalized and validated with the corresponding experimental evidence, considering a structure in with a POT/Fe_3_O_4_ multicore is wrapped with a polymeric shell containing the TRZ^+^TPB^−^.

POT has a central role as initiator of a charge disbalance triggering in the NP, ultimately resulting in an ion release (i.e., TRZ^+^) to the sample solution. POT presents two possible states: neutral (POT^0^) and oxidized (POT^+^). Neutral POT^0^ displays a characteristic fluorescence spectrum with two emission bands, one centered at 665 nm and another at 720 nm, when excited at 550 nm (Figure 2b, purple line). Upon illumination at 530 nm (green light) in presence of molecular oxygen, neutral POT° can be reversibly photo oxidized. This occurs because of the p‐type nature of this semiconductor material (i.e., electron‐hole pairs formation). Then, dissolved O_2_ can react with the promoted electrons to form O_2_^−^, with the subsequent formation of intermediate POT^+^/O_2_^−^ pairs along the polymer structure.^[^ ^40^, ^41^ ^]^ This leads to a decrease in the fluorescence signal resulting in the absence of two peaks characteristics of POT^0^. Hence, this fact confirmed the formation of POT^+^ (Figure 2b, gray line). It is here anticipated that the POT^0^/POT^+^ transition in the developed NPs can be monitored in the NP owing to the described fluorescence behavior.

The stabilization of oxidized POT⁺ is achieved by a lipophilic anion, such as tetraphenylborate (TPB^−^), present in the NP shell. This process has previously been demonstrated by our group.^[^ ^34^ ^]^ The POT^+^/TPB^−^ interaction ensures the maintenance of electroneutrality in the NP and facilitates the release of charged species (TRZ^+^ in this case) in a rather predictable manner. In connection to the presence of TPB^−^, TRZ⁺ serves as its counter cation. Due to its acid dissociation constant (pKa = 6.04), lipophilicity (log *P* = 3.13)^[^ ^42^ ^]^ and low mobility characteristics,^[^ ^43^ ^]^ TRZ⁺ is initially confined within the NP shell. Upon irradiation, POT undergoes partial oxidation and forms POT⁺, which is doped with TPB^−^ and ultimately promotes the migration of TRZ⁺ out of the NP structure to maintain charge neutrality. The fluorescence spectrum observed after irradiation (Figure 2c) confirmed the release of TRZ^+^, as evidenced by an increase in the fluorescence intensity in the supernatant and the appearance of a peak centered at 440 nm. This spectral change supports the expulsion of TRZ^+^ from the NP structure upon oxidation of POT^0^ to POT^+^. The use of TRZ⁺ as a counter cation is herein adopted as a proof of concept, with the possibility of adaptation for the vehicularization and release of other charged molecules of different nature and physicochemical characteristics.

Finally, the implementation of Fe_3_O_4_ provides certain advantages that enhance the NPs functionality towards advanced applications. On one hand, Fe_3_O_4_ NPs exhibit superparamagnetic properties, allowing the created composites to be reversibly magnetized in the presence of an external magnetic field. This feature is highly beneficial in drug release systems, as it enables precise control of NP movement while avoiding residual magnetism that might lead to undesired aggregations. On the other hand, Fe_3_O_4_ is a semiconductor with a bandgap of approximately 2.7 eV, which facilitates the formation of a heterojunction with POT that shifts the overall energy. Such a shift is critical for tuning the photophysical behavior of the NPs. Thus, the modified bandgap may allow for improved performance in specific applications, providing a platform for designing efficient nanocarriers. The synergistic effects of the Fe_3_O_4_‐POT heterojunction and their impact on enhancing NP transitions will be discussed in detail in the subsequent sections.

### Light‐Induced Versus Electrochemical‐Induced Release of TRZ^+^ from POT/Fe_3_O_4_/TRZ^+^TPB^−^ NPs

Light‐induced release of TRZ^+^ from POT/Fe_3_O_4_/TRZ^+^TPB^−^ NPs was initially demonstrated with bulk fluorescence experiments, using a 530 nm LED with an excitation power of 27.6 mW focused directly on a fix volume of the NP sample (5 mL). The sample was prepared at pH = 7.4 as detailed in the Section 3, Figure S2, from a dispersion of 200 ± 20 millions of NPs per mL. Several aliquots of the NP sample were taken before and after irradiation, and the fluorescence was measured at 550 nm to assess the oxidation of POT. In other aliquots, and after magnetic separation of the NPs, the fluorescence of the supernatant was measured at 320 nm to confirm the presence of TRZ due to its release from the NPs.

**Figure 3:**
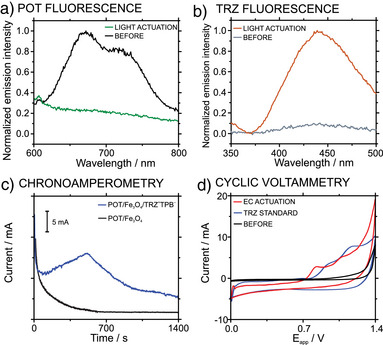
a) Fluorescence spectra of POT before (black line), and after light‐induced actuation (green line, *λ*_ex_ = 530 nm; P_ex_ = 27.6 mW). b) Fluorescence spectra of TRZ^+^ in the supernatant before (gray line) and after light‐induced actuation (orange line, *λ*_ex_ = 530 nm; *P*_ex_ = 27.6 mW). c) Chronoamperometry of POT/Fe_3_O_4_/TRZ^+^TPB^−^ NPs (blue line) and POT/Fe_3_O_4_ NPs (black line) dispersion (*E*_applied_ = 1.5 V) under agitation 600 rpm. d) Cyclic voltammetry of the solutions before (black line) and after (red line) electrochemical‐induced actuation as well as that observed for a trazodone standard solution of 2 mM concentration (blue line). EC = electrochemical.

The POT fluorescence spectra before and after light input application are depicted in Figure 3a (black and green curves). As observed, the fluorescence bands at 665 and 720 nm related to POT^0^ are present only before the light application. Effectively, the disappearance of these evidenced the presence of POT^+^ in the NPs upon light activation, which should coincide with the release of TRZ^+^ into the solution. This was confirmed with the results presented in Figure 3b (gray and orange curves), revealing a band at 440 nm only in the case of light activation in relation to the presence of TRZ in the solution but not in the NPs. In principle, these experiments demonstrated the oxidation of POT in the NPs and the concomitant presence of TRZ in the solution upon its release from the NPs after light activation. Notably, since the TRZ measurements were performed after removing the NPs from the sample, the fluorescence is unequivocally attributed to the released TRZ. In addition, we considered whether Förster Resonance Energy Transfer (FRET) between POT and TRZ could interfere with the fluorescence quantification. Spectra of POT, TRZ, and their mixture were recorded. No spectral changes or quenching effects were observed, confirming that no significant FRET occurs under the selected experimental conditions (Section 4, Figure S3). Therefore, the TRZ fluorescence signal used for quantification is not affected by energy transfer processes.

Being aware that POT can also be oxidized electrochemically but following a different mechanism than that in the light‐driven process, we additionally studied the electrochemical‐induced release of TRZ^+^ from POT/Fe_3_O_4_/TRZ^+^TPB^−^ NPs in KNO_3_ solution. Details on the experimental protocol and setup are provided in the Section 5 and Figure S4. The NP sample was subjected to a constant electric field of 1.5 V for 20 min under stirring. In principle, this potential is higher than that already established in regular electrochemical experiments for POT oxidation.^[^ ^44^ ^]^ Figure S5 (purple and blue curves) present the spectra at 550 and 320 nm in relation to the presence of POT^0^ in the NPs and TRZ in the solution, respectively. The bands related to the presence of POT^0^ decreased with respect to the control experiment (no potential application), confirming the oxidation of POT in the NP. Then, the presence of TRZ in the sample was evident after the electrochemical‐induced release.

Figure 3c (blue curve) displays the chronoamperometric response of the POT/Fe_3_O_4_/TRZ^+^TPB^−^ NPs and POT/Fe_3_O_4_ NPs (without the TRZ‐TPB complex, control NPs). A transition peak appeared at around 360 s only when the TRZ‐TPB was present. Accordingly, this must relate to the TRZ^+^ transfer from the NP to the bulk solution, which occurred in within the 50–650 s time window. To further correlate the chronoamperometric signal with TRZ^+^ molecules being released from the NPs and considering that TRZ^+^ has two irreversible oxidation peaks around 0.8 and 1.1 V (Figure 3d, blue curve), CVs before and after the electrochemical actuation were recorded. No appreciable signal was observed in the CV carried out before applying the actuation (Figure 3d, black line); whereas two oxidation peaks are noted because of the actuation (Figure 3d, red line). Compared to the CV of a standard solution of TRZ (2 mM, Figure 3d, blue line), these two peaks are ascribed to the presence of TRZ^+^. Accordingly, this experiment univocally proves the presence of TRZ in the solution and its electrochemical‐induced release from the NPs.

Note that a shift to lower potentials (∼100 mV) was observed when comparing the voltammetric peaks of the TRZ standard solution with the NP dispersion. Indeed, the dispersion system may contribute either to a displacement of the reference potential provided by the single junction reference electrode and/or a change in the overpotential magnitude of the overall process, so that this shift is expected in principle.

Although the comparison of both driven forces (light versus electrochemistry) was not straightforward, since they were investigated in different mediums as required per the different input natures (pH = 7.4 versus KNO_3_ background), we attempted to rationalize some insights. Comparing first the evolution of the oxidation state of the POT fraction after electrochemical‐induced and light‐induced release (*n* = 3), the fluorescence of POT decreases up to 50% of the original value when the NPs are directly oxidized on the electrode, while the signal completely disappeared in the case of light‐induced oxidation. There are several possible reasons: the different mechanisms expected for a direct oxidation compared to a light‐induced oxidation by using O_2_ as an electron acceptor but also, how the distribution of the POT^+^ in the NP’ structure may impact the restriction of intramolecular motion (RIM) effect, which is the main factor contributing to the modulation of the POT fluorescence.^[^ ^45^ ^]^

Having different oxidation mechanisms, it may happen that the POT structure that is finally reached in each case provides different fluorescence properties and hence, the results are not fully comparable. If this fact is obviated, it could be concluded that a higher POT fraction is oxidized by light than with the electrochemical protocol. However, the fluorescence displayed by the TRZ was higher in the case of electrochemical oxidation than the light‐driven process, which seems to contradict the behavior described for POT. Notably, it is known that the TRZ fluorescence strongly depends on the medium but also its oxidation state.^[^ ^46^, ^47^ ^]^ As such, it is difficult to establish which procedure, light‐ or electrochemical‐driven TRZ release, is more efficient in terms of TRZ^+^ delivered to the sample from the NPs.

Despite both actuation protocols demonstrating successfully releasing TRZ^+^ from the POT/Fe_3_O_4_/TRZ^+^TPB^−^ NPs, there are some cons related to the electrochemical‐induced approach that make the light‐driven strategy more appropriate for certain applications. Firstly, the required potential to ensure POT oxidation is rather high (ca. 1.5 V), with the risk for unwanted reactions occurring in parallel, e.g., water splitting or TRZ oxidation. Indeed, a gradual decrease in the TRZ^+^ signal was observed after ∼10 min of electrochemical actuation (Figure 3d), indicating that TRZ degradation takes place under these conditions. This limitation highlights that, while electrochemical oxidation confirms the redox activity of the system, it is not an ideal route for drug release.

On the contrary, light‐actuation does not induce any significant degradation of the TRZ in the given conditions, according to the electrochemical signal of the light‐delivered TRZ compared to a standard solution (Section 6, Figure S6). Slight changes were found in the peak currents together with non‐significant peak shifts. These small differences in the TRZ electrochemical behavior before and after being irradiated are consistent with normal electrode variability and non‐compensated resistance variations. Secondly, the electric field decays with the square root of distance and hence, to efficiently oxidize the NPs, the experiments need to be performed under stirring, which is not feasible in a real drug release scenario. Finally, continuing with the consideration of a real scenario, the electrodes must be implanted close to the region of interest for the TRZ delivery, which would be invasive and would not allow modification of the area of application according to clinical needs. Therefore, light‐induced release is a safe, minimally invasive, and versatile alternative and it will be explored in‐depth for the purpose of this manuscript.

### Experimental Evidence of the Mechanism Conceived for the TRZ^+^ Release From POT/Fe_3_O_4_/TRZ^+^TPB^−^ NPs

**Figure 4:**
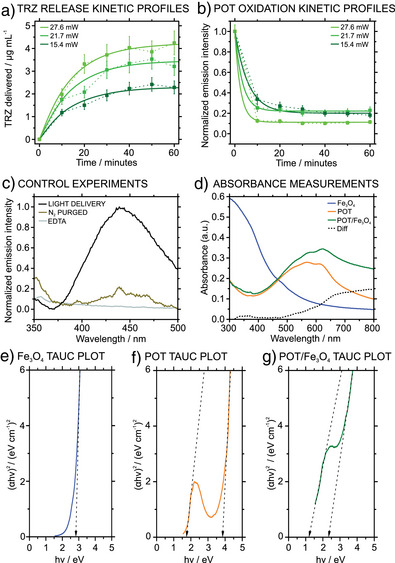
a) Release profile over time for TRZ at various irradiation intensities fitted to a pseudo‐first order reaction equation. b) Oxidation of POT over time under different irradiation intensities fitted to an exponential decay equation. c) Release of TRZ at 530 nm irradiation for 30 min (black line), experiment in N_2_‐purged solutions (brown line), and in presence of 1 mM EDTA (gray line). d) Absorbance spectra of Fe_3_O_4_ (blue), POT (orange) and POT/Fe_3_O_4_ NPs (green). The difference between the POT and POT/Fe_3_O_4_ is indicated with a dotted line. e)–g) Tauc plots of Fe_3_O_4_, POT and POT/Fe_3_O_4_ NPs, respectively. The results reported the average of triplicate measurements.

Kinetics studies were performed to understand the mechanism underlying the light‐induced release of TRZ^+^ from POT/Fe_3_O_4_/TRZ^+^TPB^−^ NPs. Using a 530‐nm illumination light, the optimization of which is shown in the next section, at three irradiation power levels, we recorded the fluorescence signal of TRZ^+^ in the supernatant within 10 min intervals over 1 h. As shown in the Figure 4a, increasing the irradiation power resulted in a higher concentration of delivered TRZ^+^, eventually reaching a steady state from the 40 min regardless of the used power. Notably, the TRZ concentration was calculated from the fluorescence measurements by means of an external calibration (Section 7, Figure S7).

The release process closely followed a kinetic profile described by the equation Q_t_ = Q_e_(1 − e^−kt^), with an excellent fit (*R* ^2^ = 0.9995), being *Q_t_* the cumulative released concentration of TRZ, *Q_e_* the concentration of TRZ in the equilibrium after the drug release process, *t* the time expressed in minutes, and *k* the rate constant of the release of TRZ^+^ expressed in min^−1^. The rate constant was estimated as *k* = 0.0704 min^−1^ by iteratively minimizing the root mean square error between the fitting and the experimental data. Several insights can be extracted about the release of TRZ following the photooxidation process of POT from this model: 1) the release follows a pseudo‐first order kinetics, meaning that the rate is high in the beginning (when the concentration inside the NPs is high) and gradually slows down as the concentration of TRZ inside the nanoparticles decreases; 2) there is an equilibrium point at which the concentration of TRZ inside the NPs remains constant. This can be displaced by modifying the irradiation intensity (see below); 3) the maximum amount of TRZ^+^ to be released at given experimental conditions is fixed and indicated by *Q_e_*.

In addition, the fluorescence decay of POT was monitored during the release. As depicted in Figure 4b, the fluorescence of POT rapidly decreased following an exponential decay. The decay was quite fast, with a halftime ranging from 2 to 4 min depending on the irradiation intensity. This is in line with previous reports that suggested a relatively fast decay of the fluorescence of similar thiophenes derivatives such as poly(3‐hexylthiophene) (PH3T) under light activation.^[^ ^48^ ^]^ Moreover, our results indicated that the TRZ release process is limited by the migration rate of the molecule from the NP to the bulk sample solution rather than the activation of the POT to form POT^+^ subunits (including its doping with the TPB^−^).

The TRZ^+^ delivery from the NP is in line with the formation of a POT/Fe_3_O_4_ heterojunction in the core of the NP. Upon illumination, positively charged holes (h^+^) and readily available electrons (e^−^) are formed. The holes can further act as charge carriers to form POT^+^, whereas the electrons react with dissolved oxygen to form superoxide, acting as an electron acceptor. The role of electrons and positive holes was further investigated by performing TRZ^+^ release experiments in presence of 1 mM ethylenediaminetetraacetic acid (EDTA) (Figure 4c, gray line) and in solutions purged with N_2_ (Figure 4c, brown line). Expectedly, if a hole‐scavenger such as EDTA is added to the solution, the release of TRZ^+^ must be hindered, demonstrating the key role of holes’ generation in the release process. Furthermore, if molecular oxygen, the main electron acceptor in the oxidation process, is removed from the solution, the release of TRZ^+^ must be hindered, as charge separation cannot be achieved any longer. Effectively, under both conditions, the fluorescence intensity of TRZ^+^ in the supernatant does not increase, further supporting the conclusion that a mechanism based on a charge carrier mediation specifically involving h^+^ and e^—^ is crucial for the TRZ^+^ release.

The described heterojunction was further investigated with absorbance experiments. First, the absorption spectra of the Fe_3_O_4_, POT and POT‐Fe_3_O_4_ NPs were obtained (Figure 4d). The spectrum of POT/Fe_3_O_4_ NPs (green line) showed a red shift compared to the spectrum of POT NPs (orange line), especially in the 0–0 transition (ca. 620 nm) associated with the crystalline domains of polythiophenes. For clarity, the difference between the modified and the unmodified POT NPs is depicted as a dotted line. This kind of red shift of the 0–0 transition has been reported to be related to the formation of an organic‐inorganic heterojunction that allows to increase the fill factor in thiophene‐based solar cells, because it affects the electronic structure of the composite.^[^ ^49^ ^]^

To better understand this interaction at the electronic level, in the case of the NPs here developed, Tauc plots were plotted and represented in Figure 4e–g. Then, to determine the energy of the direct allowed transitions of the different materials, (αhν)^2^ was plotted against the photon energy (hν). The linear part of the plot was extrapolated to (αhν)^2^ and the obtained photon energy was attributed to the different bandgaps. Fe_3_O_4_ displayed a bandgap of 2.8 eV, whereas POT presented two bandgaps, one of 1.7 eV and another of 3.8 eV. These values well agree with the literature.^[^ ^50^ ^]^ When the POT/Fe_3_O_4_ NPs are formed, the apparent energy for the two transitions in the POT displayed a red shift to 1.2 and 2.3 eV, respectively. Importantly, by shifting the more energetic transition from 3.8 eV (320 nm) to 2.3 eV (540 nm), the light‐activation operation of the developed NPs needed to generate the POT^+^ can be conducted at a biologically safe wavelength, while ultraviolet irradiation is commonly considered not suitable for in vivo applications. Overall, these experiments confirmed the formation of a heterojunction in the core of the developed NPs, which makes them useful from a practical point of view. Effectively, we can take advantage of the high‐energy bandgap of POT for the operation, which is not commonly used due to its high energy requirement.

**Figure 5:**
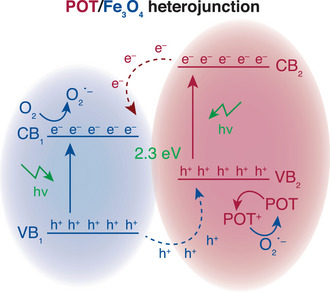
Energy level diagram of the POT/Fe_3_O_4_ heterojunction. CB: Conduction band; VB: Valence band; e^−^: electrons; h^+^: holes.

In principle, the promotion of electrons from the valence band to the conduction band is allowed through electronic interactions with the Fe_3_O_4_ cores. When the release of TRZ is monitored with fluorescence in POT/TRZ^+^TPB^−^ and POT/Fe_3_O_4_/TRZ^+^TPB^−^ NPs (Section 8, Figure S8A), it is five times higher in the presence of the heterojunction at the explored wavelength, confirming the key role of the developed multi‐core. Control experiments further support this conclusion: while POT and POT/Fe_3_O_4_ NPs without the TRZ^+^TPB^−^ complex showed no evidence of photooxidation, the presence of TRZ^+^TPB^−^ enabled the photooxidation of POT upon light illumination (Figure S8B). With all the collected experimental evidence, the suggested mechanism for the light‐activation release of TRZ is depicted in Figure 5. The involved steps can be summarized as: 1) Electrons are promoted from the valence band to the conduction band of the POT/Fe_3_O_4_ generating free electrons (e^−^) and holes (h^+^). 2) Molecular oxygen dissolved in the solution can accept the electrons and oxidize to O_2_^·–^. 3) POT can then be oxidized from POT^0^ to POT^+^ by combining its electrons with the positive holes. 4) POT^+^ may react with the O_2_^·–^ in the vicinity to form back neutral POT^0^. 5) Finally, the oxidized POT^+^ fraction encompasses the release of the charged trazodone (TRZ^+^) from the nanoparticle to the surroundings (bulk sample). These steps are expressed in the form of Equations (1)–(5) as follows:

(1) $$POT\to h^{+}+e^{-}$$

(2) $$e^{-}+O_{2}\to O_{2}^{\cdot -}$$

(3) $$h^{+}+POT\to POT^{+}$$

(4) $$POT^{+}+O_{2}^{\cdot -}\to POT$$

(5) $${\left[POTTRZ^{+}TPB^{-}\right]}_{NP}\to {\left[POT^{+}TPB^{-}\right]}_{NP}+TRZ^{+}$$

Steps (3) and (4) establish a dynamic equilibrium of the POT/POT^+^ pair that will be mainly displaced by the kinetics of the two semi‐equations that are dependent on the irradiation intensity (Equations 3 and 4). In principle, this equilibrium explains the dependence of the delivered TRZ concentration with the irradiation intensity. By performing certain control experiments, we confirmed the role of dissolved oxygen and whole generation in reactions (2) and (3), together with the importance of tuning the bandgap of the composite. Also, we could confirm that the introduction of Fe_3_O_4_ was mandatory to obtain a functional nanocarrier for the release of TRZ^+^ due to the organic‐inorganic heterojunction between both materials and the subsequent energy shift of the composites’ bandgap.

### Effect of the Irradiation Time and Wavelength on Light‐Induced Actuation and Release Performance in Real Samples

**Figure 6:**
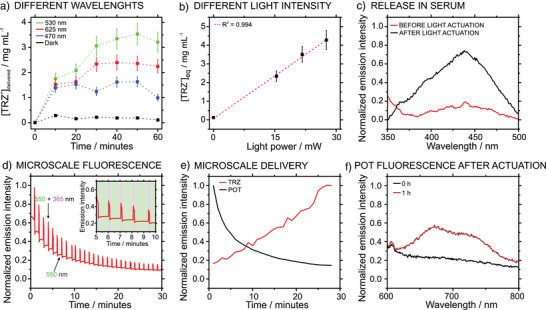
a) Effect of the wavelength (*P*_ex_ = 21.7 mW) on the release of TRZ^+^ from POT/Fe_3_O_4_/TRZ^+^TPB^−^ NPs. b) Effect of the power intensity on the release of TRZ^+^ from POT/Fe_3_O_4_/TRZ^+^TPB^−^ NPs. c) Release of TRZ^+^ in serum (*λ*_ex_ = 530 nm; *P*_ex_ = 21.7 mW). d) Microscale fluorescence measurements of POT (550 nm) and POT + TRZ (550 + 365 nm). e) Evolution over time of the TRZ release at the microscale. f) Evolution of POT fluorescence after the release process.

A series of experiments were conducted to assess the capabilities of POT/Fe_3_O_4_/TRZ^+^TPB^−^ NPs as a drug release nanoplatform. First, different wavelengths were explored. As observed in Figure 6a, TRZ^+^ light‐induced release was noted while irradiating at different wavelengths in the visible range with a constant irradiation power (*P*_ex_ = 21.7 mW). Irradiation at *λ*_ex_ = 530 nm provided the highest release: 3.5 ± 0.4 µg mL^−1^ (*n* = 3) as calculated via external calibration. This is in principle expected, since the bandgap energy observed for the POT‐Fe_3_O_4_ composite due to the heterojunctions is 540 nm, because of the energy shifting in 2.3 eV. Loading and delivery efficiency estimations are shown in the Section 9 (Tables S2 and S3). For POT/Fe_3_O_4_/TRZ^+^ TPB^−^ NPs studied in this work, the calculated loading efficiency was about 86.9%. Next, TRZ release at *λ*_ex_ = 625 nm was studied, with a maximum release of 2.4 ± 0.3 µg mL^−1.^ (*n* = 3). This moderated release agrees with the high light absorption that was recorded (see Figure 4g), even higher than that at 530 nm, which can in turn promote the separation of holes and electrons through the secondary bandgap at lower energy (1.2 eV). The irradiation at a lower wavelength (higher energy) does not necessarily induce the liberation of higher concentration of TRZ^+^. Thus, in the case of *λ*_ex_ = 470 nm, TRZ^+^ release was about 1.6 ± 0.2 µg mL^−1^ (*n* = 3). As observed in the absorption spectra previously presented (Figure 4d, green line), POT/Fe_3_O_4_/TRZ^+^TPB^−^ NPs displayed low absorption in the 470 nm range. This diminishes the release efficiency, as less photons can interact and generate electron‐hole pairs, even if the photons surpass the energy threshold imposed by the bandgap energy.

As previously discussed, by increasing the irradiation intensity, the concentration of released TRZ^+^ also increased. Notably, a higher excitation power does not only increase the release rate, but it also displaces the TRZ^+^ equilibrium concentration (Equation 5). Consequently, it is possible to tailor the drug dose by modifying the irradiation intensity, which is a disruptive approach that arises from the physicochemical properties of the herein developed nanocarrier platform, since the overall process relies on the dynamic equilibrium of the species in the organic‐aqueous interphase. Figure 6b displays the relationship between the concentration of released TRZ^+^ and the irradiation power, which was found to be linear with a rather good coefficient of determination (*R* ^2^ = 0.994). This adds further value to the reported mechanism, as the TRZ dose is directly dependent on the irradiation power, hence it can be easily adjusted according to clinical needs. Notably, according to the literature, POT is stable under irradiation within the solar intensity range (∼100 mW cm^−2^) for days to months.^[^ ^51^ ^]^ Accordingly, the conditions herein used are expected to involve from none to negligible degradation of the POT fraction in the nanoparticles.

To demonstrate the applicability of the reported mechanism as a viable drug release platform, the release capabilities were evaluated in relevant biological media. Figure 6c, displays the fluorescence related to the TRZ^+^ released into a serum sample to which the NPs were added (*P*_ex_ = 21.7 mW, *t* = 30 min). As observed, there is a high performance of the NPs due to increasing fluorescence intensity of TRZ^+^ in the supernatant. The results were compared with the release generated in deionized (DI) water: The concentrations of released TRZ^+^ were 3.2 ± 0.4 µg mL^−1^ (*n* = 3) and 3.5 ± 0.4 µg mL^−1^ (*n* = 3) for serum and DI water respectively. Thus, the release in serum represented a variation of less than 9%, which was in the range of the standard deviation for each measurement (ca. 10% of variation), confirming hence the absence of matrix effect form the complex biological media used. These results evidenced the versatility of POT/Fe_3_O_4_/TRZ^+^TPB^−^ NPs as a drug release nanoplatform, opening new venues for the testing in complex media close to the real operation conditions. Importantly, the maximum released concentration (3.5 µg mL^−1^) exceeds the reported therapeutic range of trazodone in plasma (700–1000 ng mL^−1^),^[^ ^52^ ^]^ confirming that the nanocarrier can deliver clinically relevant doses under the tested conditions upon NPs tuning.

It is noteworthy that the experiments shown Figure 6a–c were performed by measuring in the bulk sample, recording the fluorescence spectra of the different supernatants after the light‐induced release (as detailed in Section 2 and Figure S1). Furthermore, to acquire in‐situ and real‐time information at the microscale level on the TRZ^+^ release process, the POT/Fe_3_O_4_/TRZ^+^TPB^−^ NPs (10 µL of the dispersion) were irradiated at 530 nm and their fluorescence at 550 nm (POT signal) and 365 nm (TRZ signal) were recorded. The experimental details are provided in Section 10 and Figure S9. As observed in Figure 6d (see Figure S10 for the corresponding images), the fluorescence of the POT/Fe_3_O_4_/TRZ^+^TPB^−^ NPs decreased after the light‐activation release process, which is attributed to the oxidation of POT into POT^+^ and the release of TRZ^+^ from the NP structure into the surrounding medium. The lower fluorescence regions in the graph (550 nm, green regions) represent the fluorescence signal of POT only, while the periodic peaks (550 + 365 nm, purple regions) acquired every minute correspond to the combined fluorescence signals of TRZ^+^ and POT. The experiments involved fluorescence data acquisition at *λ_em_* = 550 nm (F_POT_) and *λ*_em_ = 550 nm + 365 nm (F_POT+TRZ,NP_).

In Figure 6e, the fluorescence at 550 nm (POT) is directly represented, whereas the fluorescence related to released TRZ^+^ was estimated as F_TRZ,rel_ = 1/(F_POT + TRZ, NP_ − F_POT_). In essence, since the fluorescence signals of both TRZ^+^ and POT are combined in this system, the fluorescence contribution of POT was subtracted from the total fluorescence signal to determine the amount of TRZ^+^ delivered from the NP. Then, to illustrate the release of TRZ^+^, the inverse of the fluorescence signal was plotted. As observed, the POT fluorescence (black line) decreased due to the oxidation of the POT^0^ to POT^+^, whereas the TRZ^+^ fluorescence intensity increased (red line). The results confirmed that TRZ^+^ release in the microscale follows a similar profile as that in bulk measurements. Accordingly, the bulk experiments can be considered as representative of the processes in the microscale.

Finally, the release in a complex media was also tested with the microscope. A recovery experiment was carried out noting that after being irradiated, the NPs can partly recover their native fluorescence, as can be realized in Figure 6f (experiment after 1 h). We hypothesized that this fluorescence recovery implied the formation of a TPB‐cation complex in the NP structure, with the cation being whatever present in the sample solution. Effectively, TPB‐ can form a complex with any cation in the surrounding medium (e.g., tris cation). Nonetheless, due to the lack of any ion‐selective molecule (i.e., selective ion receptors) in the NPs, a more mobile cation (e.g., lipophilic ones) present in the sample solution is more likely to be introduced in the NPs instead of the bulkier and less mobile TRZ^+^. This was confirmed by the constant TRZ^+^ concentration observed in the supernatant after irradiation: it remains constant in the solution 1 h after the light‐activation release (see Section 11, Figure S11). Overall, it was demonstrated that even if the POT oxidation process is reversible, the spontaneous reduction of the POT^+^ core to POT^0^ does not compete with the drug release process, ensuring that the released drug will not be taken up by the remaining NPs.

Additional irradiation/darkness cycling experiments were performed to assess the reversibility of the release. Unlike our previously reported K⁺‐selective nanoparticles, where the presence of an ionophore enabled a dynamic uptake/release equilibrium, the TRZ‐loaded NPs were designed for irreversible drug delivery. Upon irradiation, the POT fraction is oxidized to POT⁺ and TRZ⁺ is released, but no reincorporation of TRZ⁺ was registered during the dark periods. This confirmed that the engineered system provides irreversible photoactivation, ensuring efficient and unidirectional drug release (Section 12, Figure S12).

## Conclusion

Herein, POT/Fe_3_O_4_ nanoparticles were developed as a novel platform for the release of charged therapeutic molecules. The photooxidation of POT is utilized to enhance the release of a charged model drug that is included into the NP structure. Furthermore, Fe_3_O_4_ NPs were incorporated in the core, providing the final material with two important properties. First, superparamagnetic capabilities allow the nanoparticles to be concentrated and directed to the region of interest without inducing permanent magnetization, which could otherwise affect the stability of the nanoparticle dispersion. Second, Fe_3_O_4_ assists in customizing the optical bandgap of the material. Through the introduction of a semiconductor also in the core, the optical bandgap of the final composite is modified, allowing to utilize a low‐energy bandgap with high absorption in a biologically safe energy range. The proposed system demonstrated the delivery of the desired dose of a cationic drug either by light‐induced photooxidation or direct electrochemical oxidation. Due to the unique release mechanism, involving a dynamic equilibrium between the oxidized POT^+^ and the generated O_2_^−^, the irradiation density can shift the oxidized fraction in the equilibrium. This grants POT/Fe_3_O_4_ another functionality, as the released dose can be adapted according to the clinical needs and limitations. Overall, the developed concept offers multiple advantages, such as ease of preparation and adaptability through the incorporation of different functional materials and drugs. We expect this mechanism to open new venues for the encapsulation and release of other charged species with relevance in the biomedical field. Future efforts will be devoted to the magnetic actuation of the light‐induced drug release nanocomposites to the region of interest to perform on‐demand liberation of relevant therapeutic molecules.

## Supporting Information

The authors have cited additional references within the Supporting Information.^[^ ^53^, ^54^ ^]^

## Conflict of Interests

The authors declare no conflict of interest.

## Supporting information



Supplementary Information

Supplementary Information

## Data Availability

The data that support the findings of this study are available from the corresponding author upon reasonable request.
