# Supplementary material for: Tailored Core‐Shell Nanocarrier for Therapeutic Drug Delivery via Visible Light Activation
Source: Angew Chem Int Ed Engl. 2025 Oct 30;65(2):e202514317. doi: 10.1002/anie.202514317 (PMC12790370; doi:10.1002/anie.202514317)
Supplement: Supplementary file 1 — Supplementary Information [file ANIE-65-e202514317-s002.docx]

Supporting Information
©Wiley-VCH 2021
69451 Weinheim, Germany

Tailored Core-Shell Nanocarrier for Therapeutic Drug Delivery via Visible Light Activation

Deric Andrade-Alarcón^+[a]^, Víctor de la Asunción-Nadal^+[a]^, Gastón A. Crespo^[a, b]^ , and Maria Cuartero *^[a, b]^

[a] Deric Andrade-Alarcón; Dr. Victor, de la Asunción-Nadal; Prof. Gastón A. Crespo and Prof. Maria Cuartero
UCAM-SENS
Universidad Católica San Antonio de Murcia
UCAM HiTech, Avda. Andres Hernandez Ros 1, 30107, Murcia, Spain

[b] Prof. Gastón A. Crespo and Prof. Maria Cuartero

Department of Chemistry
KTH, The Royal Institute of Technology
Teknikringen 30, SE-100 44, Stockholm, Sweden

**Table of Contents**

[1. Experimental Procedures 2](#_Toc207322471)

[2. Characterization of the NPs 2](#_Toc207322472)

[3. Light-induced release of TRZ^+^. Bulk experiments. 3](#_Toc207322473)

[4. FRET Effect 4](#_Toc207322474)

[5. Electrochemical-induced release and determination of TRZ^+^. 4](#_Toc207322475)

[6. Irradiation effect in TRZ^+^. 5](#_Toc207322476)

[7. Calibration curve for TRZ^+^ with fluorescence readout 5](#_Toc207322477)

[8. Monitoring the release of TRZ+ 6](#_Toc207322478)

[9. Loading and delivery efficiency of TRZ^+^. Cumulative Release for each condition. 6](#_Toc207322479)

[10. Light-induced release of TRZ^+^. Microscale experiments. 7](#_Toc207322480)

[11. Delivered TRZ^+^ after release process 8](#_Toc207322481)

[12. Irradiation / darkness cycling experiment of NPs. 8](#_Toc207322482)

[13. References 8](#_Toc207322483)

1. Experimental Procedures

1.1 Reagents and materials. Aqueous solutions were prepared by dissolving the appropriate components in doubly deionized water (18.2 MΩ) (Milli Q water system, Merck Millipore). Poly(3-octylthiophene-2,5-diyl) (regioregular), iron (III) chloride (FeCl_3_), ammonium iron(II) sulfate hexahydrate (H8FeN2O8S2 · 6 H2O), sodium hydroxide (NaOH), trazodone hydrochloride (C_19_H_22_ClN_5_O·HCl), sodium tetrafluoroborate (NaBF_4_), sodium tetrakis[3,5bis(trifluoromethyl)phenyl] borate (NaTFPB, >98%), analytical grade sodium and potassium nitrate, tetrahydrofuran (THF, >99.9%), and horse serum (sterile filtered) were purchased from Sigma Aldrich and used without further purification. For the electrochemistry experiments, a glassy carbon electrode (6.09395.014, Metrohm Nordic) was used as a working electrode, a single-junction Ag/AgCl electrode (6.0733.100, Metrohm Nordic) as the reference electrode and a Pt-rod (6.0331.00, Metrohm Nordic) was used as a counter electrode.

1.2 Synthesis of the trazodone-tetraphenylborate complex To prepare the trazodone/tetraphenylborate (TRZ^+^TPB^-^) complex, a previously reported protocol was followed ^[53]^. Briefly, 25 mL of DI water containing 204 mg of trazodone hydrochloride were added dropwise to a solution containing 171 mg of sodium tetraphenylborate in 50 mL of DI water under stirring. A white precipitate was formed corresponding to the TRZ^+^TPB^-^ complex with a 1:1 stoichiometry. The white precipitate was washed with ice-cold water in a vacuum filtration system, dried at room temperature and stored at 4ºC until further use.

1.3 Synthesis of Fe_3_O_4_ NPs. Fe_3_O_4_ nanoparticles were synthesized by adapting a previously reported protocol ^[54]^. Briefly, 50 mL of DI water was bubbled using a N_2_ stream to remove the dissolved oxygen and were heated to 50ºC. Then 147 mg of (NH_4_)_2_Fe(SO_4_)_2_·6H_2_O and 65 mg of FeCl_3_·6H_2_O were added to the previous solution under vigorous stirring. 10 mL of NaOH 0.5 M were added to the solution dropwise while stirring and the resulting dispersion was sonicated in an ultrasonic bath for 60 minutes. The nanoparticles were then cleaned by magnetic separation with DI water three times. The nanoparticles were separated from the supernatant with a magnet, and the supernatant was discarded. The final Fe_3_O_4_ nanoparticles were then dried in an oven at 60ºC and stored until further use.

1.4 Synthesis of POT/Fe_3_O_4_/ TRZ^+^TPB^-^ NPs. POT/Fe_3_O_4_/ TRZ^+^TPB^-^ NPs were synthesized in a one-step emulsion method by introducing the functional materials POT and Fe_3_O_4_ NPs, the payload (TRZ^+^) and the cation exchanger (TPB^-^). Briefly, 0.8 mg of TRZ^+^TPB^-^ complex were dissolved in 1 mL THF (solution A) and 4 mg of Fe_3_O_4_ NP were dispersed in 1 mL of THF (solution B). Then 250 µL of solution A and 250 µL of solution B were added to a vial containing 5 mg of POT until the polymer is completely dissolved. This dispersion was added dropwise to a vial containing 1 mg/mL PVA in water and was stirred at 600 rpm for 30 minutes. A stream of N_2_ was blown on the surface of the solution under stirring for at least 30 minutes to completely remove all the remaining THF. The nanoparticles were removed from the supernatant by magnetic separation three times and dispersed in DI water. The clean POT/Fe_3_O_4_/ TRZ^+^TPB^-^ NPs were used directly after their preparation.

1. Characterization of the NPs

For the further characterization of the different components of the POT/Fe_3_O_4_/ TRZ^+^TPB^-^ NPs, XRD spectrum of Fe_3_O_4_ was recorded using PANalytical X’Pert PRO, FTIR of POT, PVA and POT/PVA NPs using FT/lR-6X FTIR Spectrometer with ATR PRO ONE accessory and DLS and Zeta Potential of the NPs was measured with Malvern ZETASIZER Ultra Red.


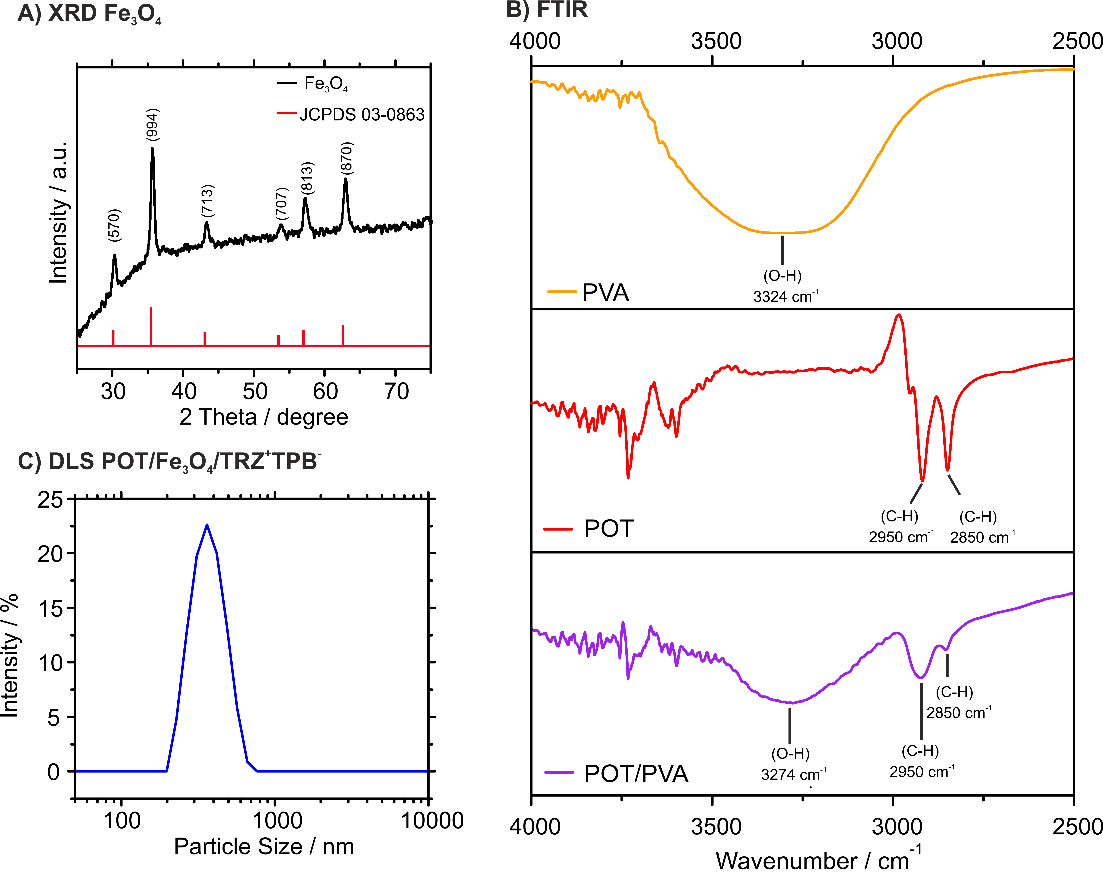


**Figure S1**. A) XRD pattern of Fe_3_O_4_ NPs. B) FTIR of PVA (orange line), POT (red line) and POT/PVA NPs (purple line) and C) DLS of POT/Fe_3_O_4_/TRZ^+^TPB^-^.

| Z-Average (nm) | 356 |
| --- | --- |
| Polydispersity Index (PI) | 0.0270 |
| Zeta Potential (mV) | -2.00 |
| Conductivity (mS/cm) | 0.0200 |

**Table S1**. Values of the particle size, polydispersity index, zeta potential and conductivity of POT/Fe_3_O_4_/TRZ^+^TPB^-^.

1. Light-induced release of TRZ^+^. Bulk experiments.

2.5 mL of POT/Fe_3_O_4_/ TRZ^+^TPB^-^ NPs were irradiated with M470L5, M530L4, MNWHL4 LED lamps, while other 2.5 mL of the dispersion were kept in dark (Figure S2). A specific time, an aliquot of 250 μL from the dispersion was taken. A Horiba FluoroMax spectrofluorometer attached to a Horiba MicroMax 384 microwell plate reader was used to record the fluorescence measurements. Then, 50 μL of POT/Fe_3_O_4_/ TRZ^+^TPB^-^ NPs were mixed with 100 μL of Tris buffer (0.1 M, pH = 7.8). We selected 550 nm as the excitation wavelength, and the fluorescence signal was recorded from 600 to 800 nm. After that, we removed the magnetic NPs using a magnet, and the 150 μL of the supernatant was measured with an excitation wavelength of 320 nm and recording the fluorescence spectrum from 350 to 500 nm. Both fluorescence signals were recorded using FluorEssence V3.9 software.


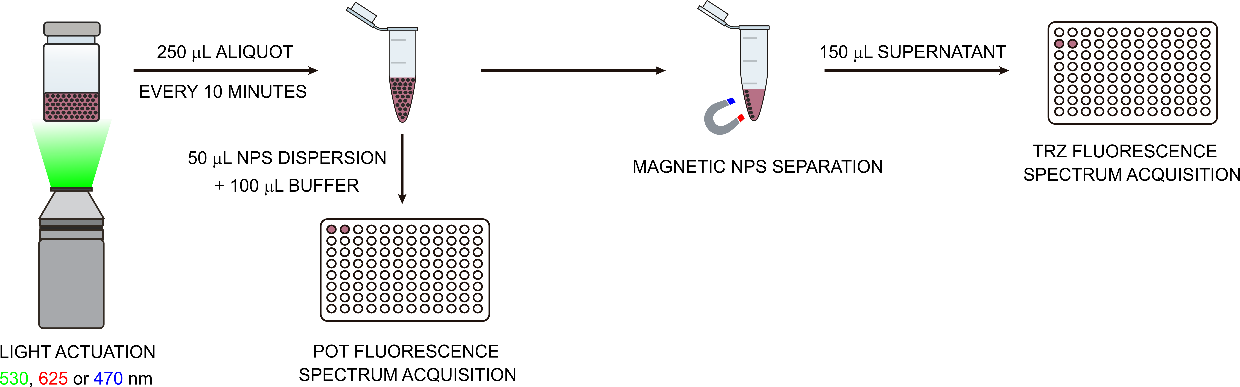


**Figure S2.** Illustration of the steps established for the sample preparation in the bulk experiments.

1. FRET Effect


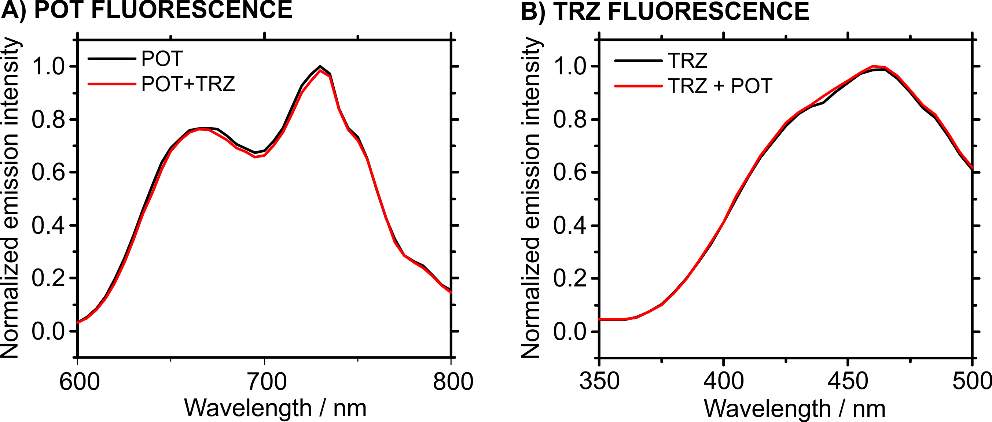


**Figure S3**. A) POT NPs fluorescence spectrum (black line) and POT NPs fluorescence spectrum in presence of TRZ^+^ (red line). B) TRZ^+^ fluorescence spectrum (black line) and TRZ^+^ fluorescence spectrum in presence of POT NPs (red line).

1. Electrochemical-induced release and determination of TRZ^+^.

Cyclic voltammetry and chronoamperometry were performed using an Autolab PGSTAT302N potentiostat. The potentiostat was controlled by Nova 2.1 software (supplied by Autolab). For all the electrochemical experiments a 3-electrode configuration of the cell was employed. A 3mm diameter glassy carbon electrode as a working electrode, a single junction Ag/AgCl as a reference electrode and a Pt rod as a counter electrode (Figure S4). To carry out the measurements POT/Fe_3_O_4_/ TRZ^+^TPB^-^ NPs were redispersed in 5 mL of 0.1 M KNO_3_ solution.

The initial presence of TRZ^+^ was investigated using cyclic voltammetry (CV) within a potential window of 0 to 1.4 V at a scan rate of 100 mVs-1. Subsequently, a chronoamperometry experiment was conducted by applying a constant potential of 1.5 V to the nanoparticle dispersion for 20 minutes under stirring at 600 rpm. Following this, a second CV was performed under the same conditions as the initial one to evaluate the delivered concentration of TRZ^+^. The experimental setup is illustrated with the image presented in Figure S4.


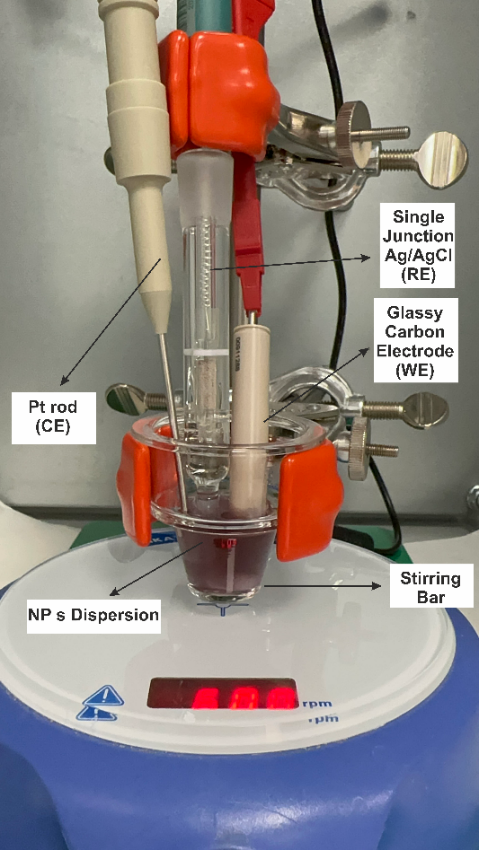


**Figure S4.** Electrochemical cell 3-electrode configuration for the electrochemical actuation experiments.


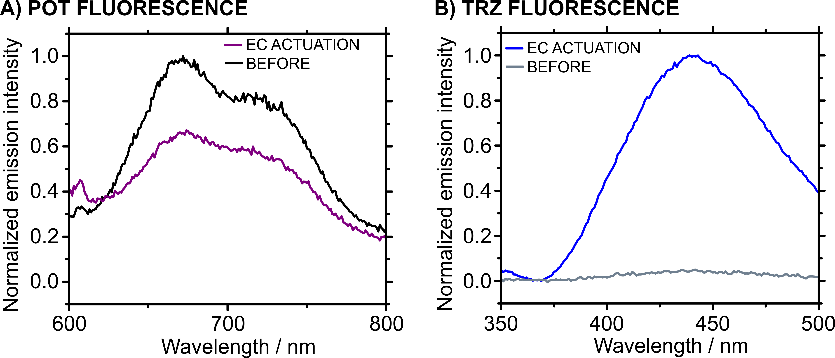


**Figure S5.** Fluorescence spectra of POT before (black line) and after EC actuation (purple line, E_applied_ = 1.5 V), B) Fluorescence spectra of TRZ^+^ in the supernatant before (grey line) and after EC actuation (blue line, E_applied_ = 1.5 V).

1. Irradiation effect in TRZ^+^.


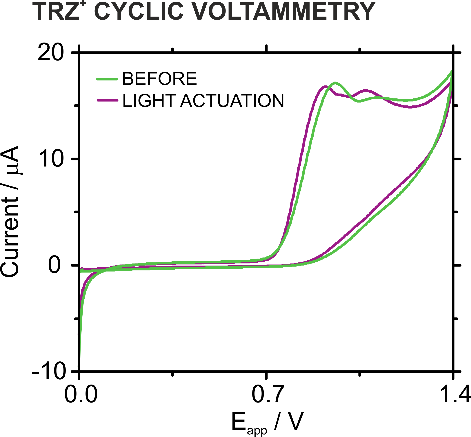


**Figure S6.** Cyclic voltammetry of independent aqueous solution of 2 mM TRZ^+^ in 0.1 M KNO_3_ before light actuation (green line) and after light-actuation (purple line) (λ_ex_ = 530 nm; P_ex_ = 21.7 mW). Scan rate 100 mV/s.

1. Calibration curve for TRZ^+^ with fluorescence readout

**
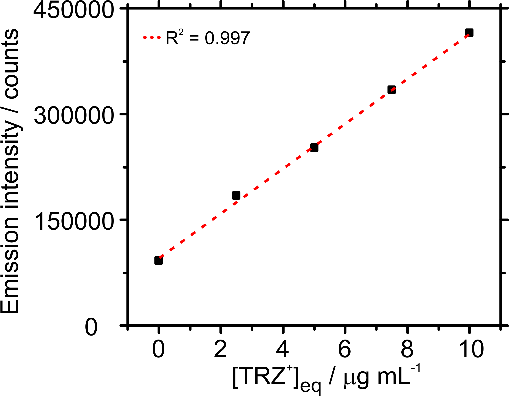
**

**Figure S7.** Calibration curve of trazodone hydrochloride.

1. Monitoring the release of TRZ+


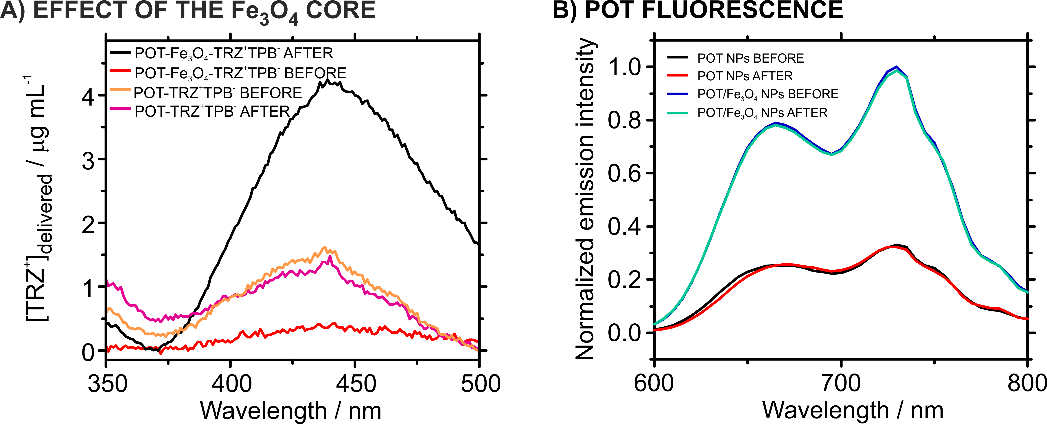


**Figure S8.** A) Release of TRZ^+^ from NPs based on a POT core before (orange line) and after (pink line) irradiation (λ_ex_ = 530 nm; P_ex_ = 21.7 mW), and NPs based on a Fe_3_O_4_/POT core before (red line) and after (black line) irradiation. B) POT NPs spectrum before light-actuation (black line) and after light-actuation (red line) and POT/Fe_3_O_4_ spectrum before (blue line) and after light-actuation (turquoise line) (λ_ex_ = 530 nm; P_ex_ = 21.7 mW).

1. Loading and delivery efficiency of TRZ^+^. Cumulative Release for each condition.

**Loading efficiency:**

$$\text{Loading Efficiency}= \frac{{[TRZ]}_{0}-{[TRZ]}_{sup}}{{[TRZ]}_{0}} \times100$$

**Delivery efficiency:**

$$\text{Delivery Efficiency}= \frac{{[TRZ]}_{delivered}}{{[TRZ]}_{load}} \times100$$

with:

- [TRZ]_0_: initial concentration of TRZ used in the preparation
- [TRZ]_sup_: concentration of TRZ remaining in the supernatant after nanoparticles preparation.
- [TRZ]_load_ = [TRZ]_0_ – [TRZ]_sup_: concentration of TRZ loaded in the particle
- [TRZ]_delivered_: concentration of TRZ released from the NPs.

For POT/Fe_3_O_4_/TRZ^+^TPB^-^ NPs studied in this work, the calculated loading efficiency was about 86.9%

Loading and delivery efficiency of TRZ^+^. Cumulative Release for each condition.

**Table S2** Percentage of cumulative release compared to the loading of TRZ+ from nanoparticles relative to loading under different irradiation wavelengths.

Different wavelength

| Time (min) | Dark | 470 nm | 530 nm | 625 nm |
| --- | --- | --- | --- | --- |
| 0 | 0 | 0 | 0 | 0 |
| 10 | 4.01 | 15.30 | 19.30 | 16.82 |
| 20 | 2.03 | 16.93 | 22.94 | 18.00 |
| 30 | 2.83 | 13.74 | 33.74 | 25.78 |
| 40 | 2.44 | 17.85 | 36.90 | 26.45 |
| 50 | 2.44 | 17.97 | 38.96 | 26.02 |
| 60 | 1.38 | 10.91 | 35.40 | 24.73 |

*All the values are expressed as percentages (%)*

**Table S3**. Percentage of cumulative release compared to the loading of TRZ+ from nanoparticles relative to loading under different light intensities.

Different light intensity

| Time (min) | Dark | 15.4 mW | 21.7 mW | 27.6 mW |
| --- | --- | --- | --- | --- |
| 0 | 0 | 0 | 0 | 0 |
| 10 | 4.01 | 13.01 | 19.30 | 21.87 |
| 20 | 2.03 | 16.57 | 22.94 | 36.61 |
| 30 | 2.83 | 21.36 | 33.74 | 41.02 |
| 40 | 2.44 | 24.65 | 36.90 | 44.49 |
| 50 | 2.44 | 26.69 | 38.96 | 42.51 |
| 60 | 1.38 | 25.22 | 35.40 | 46.84 |

*All the values are expressed as percentages (%)*

1. Light-induced release of TRZ^+^. Microscale experiments.

A Nikon Eclipse Ti2 inverted microscope was used for microscale imaging and fluorescence measurements. In the microscale fluorescence experiments, 10 μL of the NPs dispersion was added to a glass slide. A CoolLED pE-4000 was used as the excitation source. The fluorescence emission was filtered using a Semrock NKBV-0177 filter and recorded using a Nikon DS-Qi2 monochrome camera. NIS Elements BR 5.41 software was used for data acquisition and interpretation. All the measurements were repeated at least in triplicate.

To monitor both the oxidation of POT and the release of TRZ⁺ in situ, a fluorescence microscopy experiment was conducted. For this purpose, 10 μL of the TRZ-loaded nanoparticle dispersion was irradiated with a 530 nm LED built in the microscope system. During irradiation, the fluorescence signal was recorded at 665 nm Figure S9A, corresponding to the POT excitation region, to confirm nanoparticle oxidation. As shown in Figure 4D, the fluorescent intensity of POT decreases over time, indicating oxidation activation by the irradiation. Simultaneously, fluorescence at 350 nm, corresponding to the TRZ⁺ excitation region, was recorded every minute while irradiating at 530 nm to track TRZ⁺ release Figure S9B. The observed spikes in the graph indicate changes in fluorescence intensity within the nanoparticles. The decreasing fluorescence signal suggests the gradual release of TRZ⁺ from the nanoparticle structure during the irradiation process.

Particle fluorescence imaging and image analysis: To be able to record individual nanoparticles for particle counting, we constrained diluted dispersions of nanoparticles between a sample holder and a glass cover, and long exposure time (1 second) fluorescence images were recorded to increase the counts-to-noise ratio. Later, the images were processed using the ImageJ software using the watershed tool to identify individual components of the clusters and analyzed using the particle analysis tool.

**
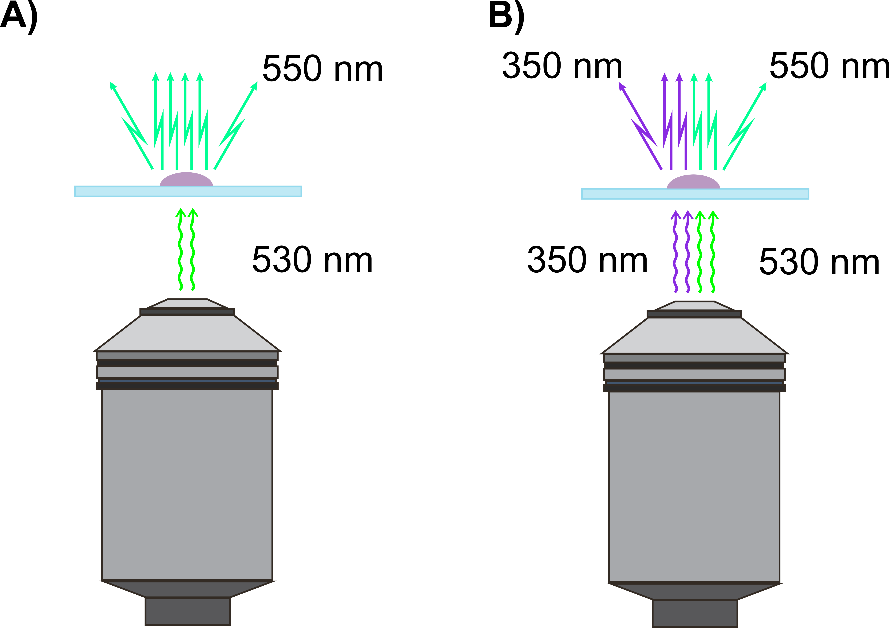
**

**Figure S9.** Scheme of microscale fluorescence experiments, with an irradiation wavelength of 530 nm. A) Fluorescence of POT (550 nm). B) Fluorescence of TRZ^+^ (350 nm) + POT (550 nm).


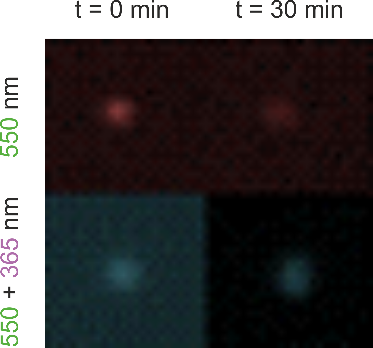


**Figure S10.** Microscope fluorescence images of single NPs at 550 nm (POT) and 550 + 365 nm (POT + TRZ^+^) at different irradiation times.

1. Delivered TRZ^+^ after release process


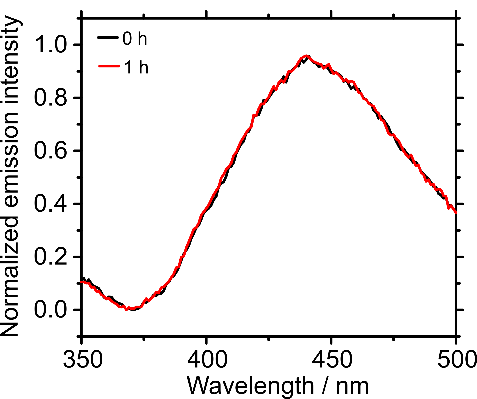


**Figure S11**. TRZ^+^ fluorescence spectra after light actuation t=0 (black line) and t=1 hour (red line)

1. Irradiation / darkness cycling experiment of NPs.


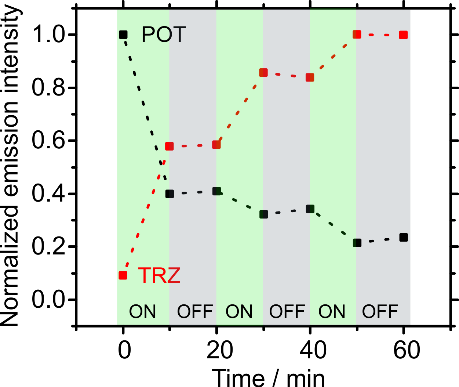


**Figure S12.** Evolution of the POT fluorescence (black line) and TRZ+ fluorescence in the supernatant (red line) in an irradiation/darkness cycling experiment.

1. References

[53] M. S. García, J. Ortuño, M. I. Albero, M. Cuartero, *Anal Bioanal Chem* 2009, *394*, 1563–1567.

[54] V. de la Asunción-Nadal, C. Franco, A. Veciana, S. Ning, A. Terzopoulou, S. Sevim, X. Z. Chen, D. Gong, J. Cai, P. D. Wendel-Garcia, B. Jurado-Sánchez, A. Escarpa, J. Puigmartí-Luis, S. Pané, *Small* 2022, *18*, 2203821.
